# Three-Dimensional Printing in Minimally Invasive Cardiac Surgery: Optimizing Surgical Planning and Education with Life-Like Models

**DOI:** 10.21470/1678-9741-2020-0409

**Published:** 2022

**Authors:** Paola Keese Montanhesi, Giselle Coelho, Sergio Augusto Fudaba Curcio, Robinson Poffo

**Affiliations:** 1 Hospital Israelita Albert Einstein, São Paulo, São Paulo, Brazil.; 2 Hospital Infantil Sabará, São Paulo, São Paulo, Brazil.; 3 EDUCSIM Institute, São Paulo, São Paulo, Brazil.

**Keywords:** Minimally Invasive Surgical Procedures, Printing, Three-Dimensional, Models, Anatomic, Preoperative Planning, Cardiac Surgical Procedures

## Abstract

Over recent years, the surgical community has demonstrated a growing interest in imaging advancements that enable more detailed and accurate preoperative diagnoses. Alongside with traditional imaging methods, three-dimensional (3-D) printing emerged as an attractive tool to complement pathology assessment and surgical planning. Minimally invasive cardiac surgery, with its wide range of challenging procedures and innovative techniques, represents an ideal territory for testing its precision, efficacy, and clinical impact. This review summarizes the available literature on 3-D printing usefulness in minimally invasive cardiac surgery, illustrated with images from a selected surgical case. As data collected demonstrates, life-like models may be a valuable adjunct tool in surgical learning, preoperative planning, and simulation, potentially adding safety to the procedure and contributing to better outcomes.

**Table:** 

Abbreviations, acronyms & symbols	
2-D	= Bidimensional
3-D	= Three-dimensional
CHD	= Congenital heart defects
CT	= Computerized tomography
MICS	= Minimally invasive cardiac surgery
MRI	= Magnetic resonance imaging

## INTRODUCTION

Advanced cardiac surgical procedures for acquired and congenital heart diseases demand accurate preoperative planning and continuous update. Heart surgeons and structural interventionalists are constantly seeking for valuable tools to better understand complex anatomy and define the best surgical approach. In that scenario, adequate preoperative evaluation incorporates multiple strategies for imaging assessment of the surgical anatomy.

Although current cardiovascular imaging modalities like computerized tomography (CT), magnetic resonance imaging (MRI), echocardiography, and post-processing softwares may provide adequate visualization of the pathology, bidimensional (2-D) view has notable limitations, and surgeons often find different anatomical arrangements in the intraoperative period.

Complex cardiovascular diseases such as congenital heart malformations can be very difficult to be fully understood in 2-D CT, MRI, or echocardiographic images^[1,2]^. Furthermore, three-dimensional (3-D) digital reconstructions may not offer proper knowledge of anatomical relations, structure sizes, and depth. The 3-D printing method has emerged as an alternative to solve this problem and to improve pathology comprehension^[3,4]^.

The 3-D printing technology was introduced by Charles Hull in 1986^[1,2]^. Since then, it has been largely applied for the production of prototypes and industrial components and, more recently, for medical purposes^[5]^. Today, print models can be crafted for several medical applications including creation of anatomy teaching tools, development of functional or deformable models for preoperative planning, and building tissue and organ structures in the field of tissue engineering^[2,5-9]^.

Printed models offer improved visualization, tactile experience, and accurate information for procedural planning of surgical reconstruction and device implantation^[4,7,8,10,11]^.For that reason, its use has increased among medical specialties, such as general surgery (for liver transplantation with living donor)^[3,7,12]^, neurosurgery (complex skull base surgeries, craniosynostosis, cerebral aneurysms)^[13-22]^, plastic surgery (prosthesis implantation, organs, and tissue reconstruction)^[23]^, vascular surgery (aneurysms)^[24]^, orthopedic surgery (repair of complex fractures)^[25-27]^, and many others^[28-36]^.

Additionally, 3-D models can be helpful as a teaching tool assisting students and surgical trainees to understand spatial anatomy, to better comprehend surgical procedures^[2,7,8,12,37]^, and to enhance cardiac critical care via simulation training of multidisciplinary intensive care teams^[3,37-39]^. Other important application is to help patients and their families to recognize the complexity of the pathology, discussing surgical planning and potential complications in detail^[38,39]^.

Particularly in cardiovascular surgery, there are many potential contributions. The 3-D printing technology may assist surgeons to plan and practice the surgical approach intended, developing strategies to deal with uncommon and high-risk intraoperative scenarios^[8,11,12,40]^. Printed aortic aneurysm models have been used in planning endovascular repairs, for example^[17,41-44]^. This tool may be especially helpful for guiding surgeons in complex intracardiac defects and multiple valve surgeries, either for preoperative planning or teaching^[3,6,45-49]^. It can also contribute to create or refine intracardiac devices^[2,50]^.

The main goals of this review are to summarize the applications of 3-D printing in cardiovascular procedures, particularly in minimally invasive cardiac surgery (MICS), to discuss potential advantages and current limitations, and to highlight its role in preoperative surgical planning and medical education.

## ILLUSTRATIVE CASE REPORT

The following case was selected to illustrate the process of creating and printing a 3-D model and the usefulness of life-like models in the surgeon’s preoperative evaluation and training.

**Fig. 1:**
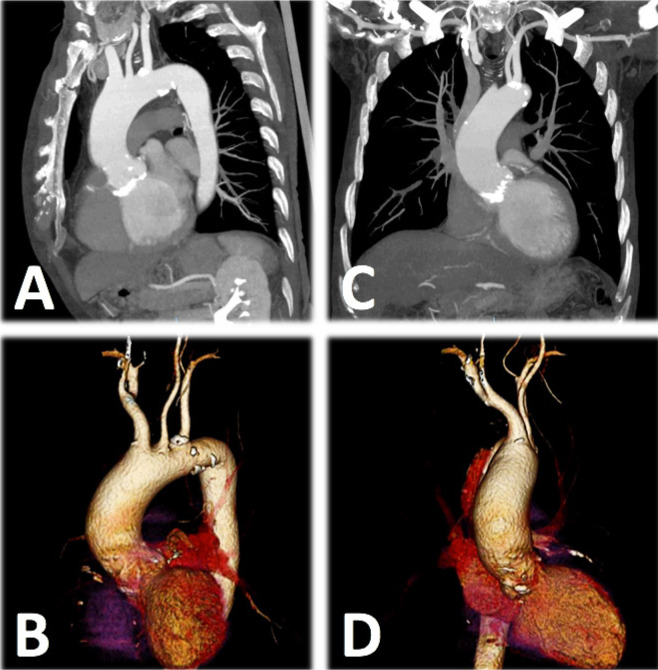
Computerized tomography angiography images of the aortic root, sinotubular junction, and ascending aorta. A) Obliquus view. B) Three-dimensional (3-D) reconstruction of the obliquus view. C) Anteroposterior view. D) 3-D reconstruction of the anteroposterior view.

A 75-year-old man with symptomatic low-flow low-gradient severe aortic stenosis due to a bicuspid aortic valve and dilation of the ascending aorta was assessed for elective minimally invasive aortic valve replacement. His left ventricular ejection fraction was 35%, and his past medical history was remarkable for hypertension, smoking, and progressive dyspnea in keeping with New York Heart Association Class III. His Society of Thoracic Surgeons mortality risk score was 2.414%. Preoperative laboratory screening, chest radiography, and cardiac angiography showed no abnormalities. CT angiography showed a severely calcified bicuspid aortic valve and dilation of the ascending aorta (43.6 × 42.8 mm), with normal aortic root and sinotubular junction (Figure 1).

**Fig. 2:**
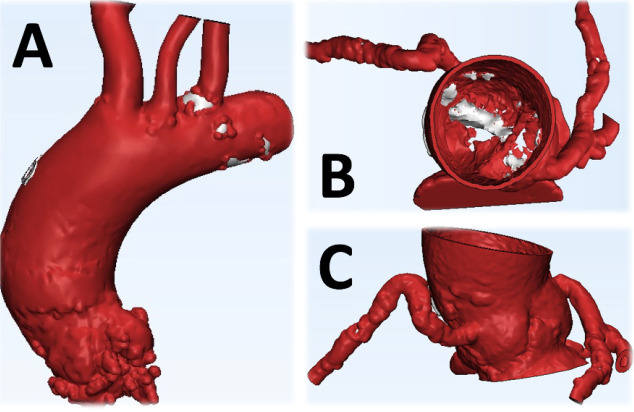
Virtual reconstruction of the target anatomy from computerized tomography angiography images (segmentation process). A) Exterior view of the aorta. B) Interior view of the aorta showing a calcified bicuspid aortic valve. C) Details of the coronary sinuses and arteries.

**Fig. 3:**
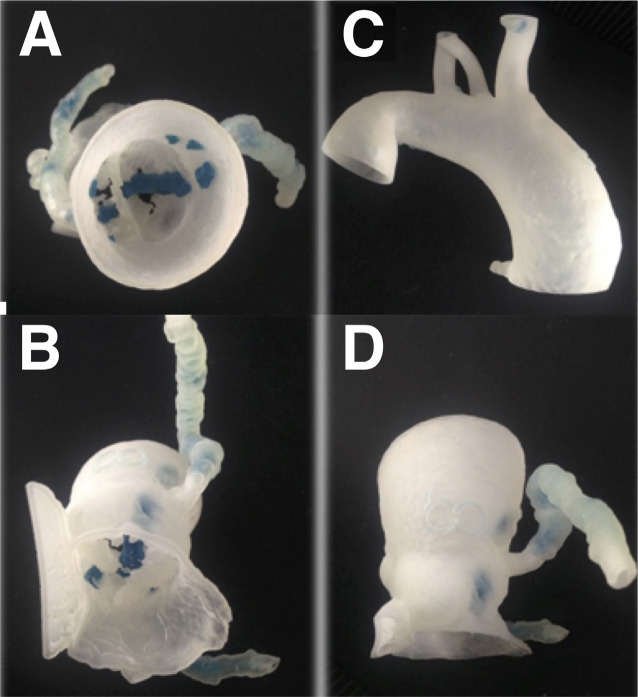
- Three-dimensional printed models for preoperative planning. Calcifications shown in blue. A) Aortic valve (axial view). B) Ventricular view of the aortic valve calcifications (blue color). C) Ascending aorta and aortic arch (posterior view). D) Aorta and coronary arteries (anterior view).

**Fig. 4:**
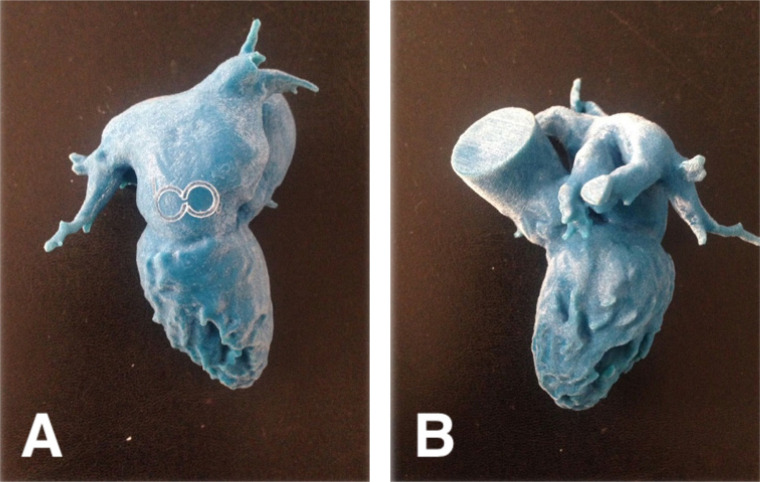
Three-dimensional printed model of the heart for preoperative planning (real-size model). A) Right lateral posterior view. B) Left lateral view.

Digital 3-D models were created from the CT angiography dataset using the Mimics® software (Materialise®, Leuven, Belgium). Data was segmented to develop a virtual model that clearly showed sizes and anatomical relations between structures, including calcification spots in the aortic valve and root and the ascending aorta dilation (Figure 2). After the segmentation process, the models were printed (Figures 3 and 4), what consists of the deposition of successive overlapping layers of material for the construction of the piece. The PolyJet 3-D technology was chosen for building this complex model as it allowed for different density materials and colors for realistic simulations. The model was printed in 0.014 mm layers and the complete process duration was 42 hours (38 hours of printing and four hours of finishing process). The anatomic model allowed a detailed discussion of the surgical approach by providing tissues of different colors, consistencies, and resistances.

The surgical team participated in the planning sessions and once the models were ready to be manipulated, the surgeons simulated surgical procedures with two different valve designs (intra-annular and supra-annular). They also decided on the minimally invasive access between L-shaped partial sternotomy and anterior thoracotomy and selected cannulation and cross-clamping strategies based on the new perception provided by the printings. Additionally, the models helped the team to foresee critical moments of the surgery. Therefore, it is the team’s unanimous perception that preoperative planning with printed models potentially saved time in the operating room, reduced potential postoperative complications, and contributed for better results.

**Fig. 5:**
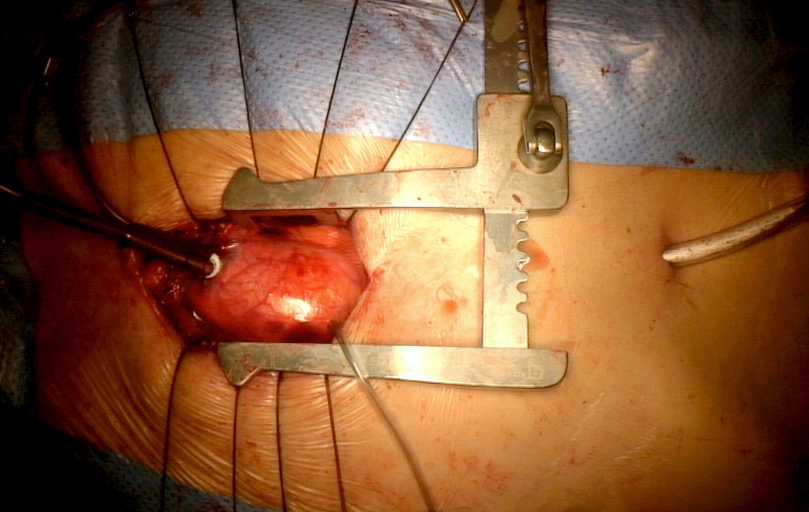
Adequate exposure of the ascending aorta following partial “L” shaped sternotomy.

**Fig. 6:**
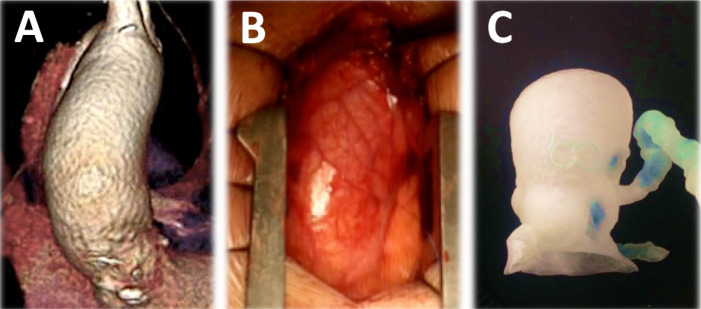
Comparison between computerized tomography angiography reconstruction (A), real anatomy (B), and printed model (C) of the dilated ascending aorta: close relation of size and shape between methods.

**Fig. 7:**
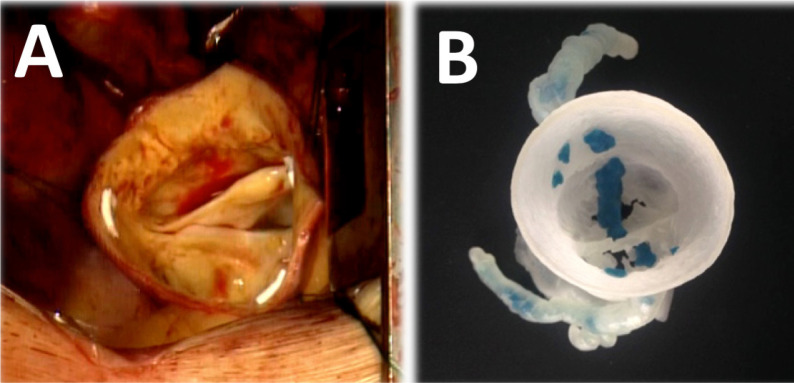
Aortic valve inspection after resecting the ascending aorta aneurysm: calcified and bicuspid aortic valve (A) adequately correlated with previous three-dimensional models (B).

The patient was submitted to minimally invasive aortic valve replacement and correction of the ascending aorta aneurysm through a partial upper L-shaped sternotomy (Figure 5). During the procedure, surgeons were able to verify a close correspondence between the 3-D models and live anatomy (Figures 6 and 7). The patient recovered well and remains asymptomatic at follow-up.

## DISCUSSION

Over recent years, the surgical community has demonstrated a growing interest in imaging advancements that enable detailed and accurate preoperative diagnoses. 3-D printing emerged as an attractive tool to complement pathology assessment and surgical planning^[50,51]^. With its wide range of challenging procedures and innovative techniques, MICS represents an ideal territory for testing its precision, efficacy, and clinical impact.

The 3-D modeling process is based on the following steps: 1) acquisition of CT imaging dataset; 2) segmentation process and creation of segmentation mask; 3) conversion of the segmentation mask into a digital 3-D patient-specific model; 4) adjustment of the digital model; and 5) 3-D printing of the multi-material model.

Traditionally, the data segmentation consists in converting anatomical information obtained by CT and cardiac MRI into a 3-D digital model that precisely replicates target anatomic structures, congenital heart defects (CHD), or vascular anomalies^[4,7,8,11,40]^. Most recently, models derived from echocardiography emerged showing technical feasibility and accuracy of < 1 mm^[1,40,52]^. Regardless of the imaging modality used, only after optimal segmentation and image postprocessing the virtual model is printed in the selected material.

Several printing processes are available: stereolithography fabricates a solid object from a photopolymeric resin using digitally guided ultraviolet laser light. Fused deposition modeling creates a 3-D structure by extruding melted thermoplastic filaments layer by layer, along with a physical support material that is later dissolved away. Selective laser melting creates strong parts of fused material or ceramic powder using a high-power laser beam and is also preferred for building functional prototypes or medical implants, such as facial bone replacements^[2,25,26]^. Last of all, the PolyJet technology creates 3-D prints through a process of jetting thin layers of liquid photopolymers that are instantly hardened using ultraviolet light. This technique can combine multiple materials and colors simultaneously, resulting in highly complex models with smooth surfaces and thin walls (down to a resolution of 0.016 mm) and it is used, among many purposes, for fabricating flexible patient-specific anatomical models with greater accuracy when compared to other printing methods.

It seems a common understanding between surgeons that printed models provide better understanding of anatomic characteristics^[4,13,23,27-29,46]^ and consequently help with preoperative planning by facilitating visualization of potential hazards and anatomic variations^[4,15,22,30-34,42]^. Similarly to our experience, many surgeons appreciated the hands-on experience provided by the physical model^[4,6,12,28,37,45,51]^. Additionally, several reports confirm the effectiveness of 3-D printing technique for preoperative planning in complex anatomies^[4,13,45,46]^ as it allows the surgical team to select more suitable implants or devices for the procedure^[16,22,35]^ and to anticipate difficulties that might appear by simulating the real surgery^[4,6,24,28,32,37,45,47,51]^. Moreover, one third of the studies showed decreased operating times and reduced risk of postoperative complications when using 3-D printing^[4,40]^. Reduced blood loss and transfusion requirements^[4]^ were also highlighted. Likewise, there was a significant reduction in patient and surgical team exposure to radiation when models were used^[4,17,18,40-44]^.

Furthermore, our illustrative case allowed for intraoperative measurement of the target anatomy and facilitated comparisons of real structures, 3-D CT reconstructions, and printed models, showing high precision. Many published studies also demonstrated that models’ accuracy was a major advantage even in complex cases^[1,6,45-49]^, and the PolyJet printing technique showed greater precision compared to other printing methods^[4,11]^. Accuracy is a key factor for patient safety, as clinical decisions are based on the 3-D printed model. Hence, it is important to integrate different imaging modalities to create highly accurate hybrid 3-D models and to engage both cardiologists and surgeons in processes of reconstruction, segmentation, and prototyping^[40]^.

According to literature, younger surgeons tend to report greater satisfaction with 3-D model manipulation than proficient ones, but all described the experience as highly beneficial^[12]^. Preoperative surgical simulation can help students, residents, expert doctors, and multidisciplinary teams to address surgical limitations by providing opportunities to practice unusual procedures and to exercise efficiently without exposing patients’ lives to unjustified risk^[2,6,11,37-39,40,51]^. Ultimately, the application of the 3-D printing technology contributes to improve patient safety by decreasing perioperative morbidity^[4,8,11,19-21,36,45]^.

Similar experience is reported among pediatric cardiac surgeons^[1,6,7,40,45-49]^. CHD are frequently complex cases that benefit from careful imaging assessment using 3-D models for better understanding anatomical defects, interactions of cardiac structures, and for planning the surgical treatment^[45,48,49,53]^. A prospective multicenter case-crossover study measured the influence of 3-D printing in CHD surgical planning by providing surgeons with printed models after a first multidisciplinary discussion and registering a possible change in surgical strategy. There was significant impact on clinical practice, with models redefining the surgical approach in 19 of 40 cases^[45]^. Models also showed high accuracy, with a mean bias of-0.27 ± 0.73 mm when compared to MRI or CT measurements. Of all the surgeons enrolled, 96% agreed or strongly agreed that printed models provided better understanding of the CHD complex morphology and helped reducing the potential for surgical complications^[45]^. In conclusion, 3-D models were considered precise replicas of the cardiovascular system and helped redefine surgical approach.

With the constant evolution of cardiovascular surgery and the development of minimally invasive techniques worldwide, new surgical skills and adjunct technologies have been incorporated for safer and less invasive procedures^[54-57]^. The potential benefits of MICS include shorter length of hospital stay, reduced bleeding and need for blood products transfusion, less pain, earlier mobilization and return to social and professional activities, better cosmesis, and, ultimately, greater patient satisfaction when compared to conventional procedures^[55-57]^. These results may be enhanced by an adequate preoperative planning, in which the addition of new tools for careful preoperative imaging diagnosis help surgeons to achieve better outcomes. Consequently, by improving surgical planning, 3-D printings have the potential to increase procedural efficiency and contribute for excellent surgical results^[53]^.

Especially in MICS, where sensory perception and surgical field exposure are limited, 3-D printed models have inherent benefits over 2-D or 3-D digital images. By providing tactile and real-size knowledge, models enhance comprehension of anatomy, depth perception, and spatial orientation’s capability. Moreover, they are portable objects easily sterilized to assist intraoperative navigation^[12]^. In association with tactile and more realistic advantages of 3-D printing, the augmented memorization of essential details may for itself be an argument in favor of using 3-D printing prior to complex surgeries. Nowadays, print models with similar biotexture to a patient’s heart are being used for simulations and training in MICS^[53]^. Future perspectives include 3-D printing for testing interventions, creating dynamic models simulating the cardiac cycle, and for building tissue and organ structures in the field of tissue engineering^[1,2,5,9,58,59]^.

Nonetheless, there are limitations for widespread use of this technology. Currently, the technology is not available in all health care centers, as few have 3-D printers,. Alongside, there are technical limitations of bedside imaging and availability of advanced imaging required to provide high resolution data (CT, CT angiography, or MRI). Also, the segmentation software has limitations in distinguishing tissues of very similar density and materials that can be manipulated — cut, dissected, retracted, sutured —, and for that reason the authors strongly believe that the involvement of the surgeon in the segmenting process is a key factor to reduce some of these limitations^[32]^. Finally, institutions that do not have a printer can buy 3-D models from specialized companies, but the relatively high cost of production may restrain its use.

Despite all 3-D printing advancements, there are no controlled studies to determine the clinical impact of print models in cardiovascular surgery. However, even in face of limited literature^[60]^, this review reinforces the promising prospects of 3-D printing. Future studies may provide scientific validation using well-defined performance measures, possibly followed by integration of this new educational tool into training and daily practice in the operating room.

## CONCLUSION

In conclusion, the use of 3-D modeling can decrease operating time and intraoperative errors, increase efficiency, and may consequently decrease liability by optimizing the surgeon’s learning curve. Nevertheless, it should not replace the traditional imaging assessment, but complement clinical judgment and surgical knowledge. In MICS, it may be a useful adjunct tool for surgical preoperative planning and simulation as it sums safety to the procedure and potentially contributes to better outcomes and to improved learning prospects.

**Table:** 

Authors' roles & responsibilities	
PKM	Substantial contributions to the conception of the work; and the acquisition of data for the work; drafting the work and revising it; agreement to be accountable for all aspects of the work in ensuring that questions related to the accuracy or integrity of any part of the work are appropriately investigated and resolved; final approval of the version to be published
GC	Substantial contributions to the conception of the work; and the interpretation of data for the work; drafting the work and revising it; agreement to be accountable for all aspects of the work in ensuring that questions related to the accuracy or integrity of any part of the work are appropriately investigated and resolved; final approval of the version to be published
SAFC	Substantial contributions to the acquisition of data for the work; agreement to be accountable for all aspects of the work in ensuring that questions related to the accuracy or integrity of any part of the work are appropriately investigated and resolved; final approval of the version to be published
RP	Substantial contributions to the conception of the work; revising the work; agreement to be accountable for all aspects of the work in ensuring that questions related to the accuracy or integrity of any part of the work are appropriately investigated and resolved; final approval of the version to be published
